# The mediatory role of inflammatory markers on the relationship between the NOVA classification system and obesity phenotypes among obese and overweight adult women: a cross-sectional study

**DOI:** 10.3389/fnut.2023.1226162

**Published:** 2023-12-11

**Authors:** Mahya Mehri Hajmir, Farideh Shiraseb, Dorsa Hosseininasab, Yasaman Aali, Shabnam Hosseini, Khadijeh Mirzaei

**Affiliations:** ^1^Students’ Scientific Center, Tehran University of Medical Sciences, Tehran, Iran; ^2^Department of Community Nutrition, School of Nutritional Sciences and Dietetics, Tehran University of Medical Sciences, Tehran, Iran; ^3^Department of Nutrition, Science and Research Branch, Islamic Azad University, Tehran, Iran; ^4^School of Human Nutrition, McGill University, Quebec, QC, Canada; ^5^Food Microbiology Research Center, Tehran University of Medical Sciences, Tehran, Iran

**Keywords:** inflammatory markers, metabolic healthy and unhealthy women, NOVA classification system, phenotypes, ultra-processed foods

## Abstract

**Background:**

Diet and inflammation both play important roles in the occurrence of obesity. We aimed to investigate the role of inflammation in the development of both metabolically healthy obese (MHO) and metabolically unhealthy obese (MUHO) individuals.

**Methods:**

This cross-sectional study included 221 overweight and obese women aged 18–56 years. The study assessed the metabolic health phenotypes of the participants using the Karelis criterion score. Additionally, dietary intakes were evaluated using a 147-item semi-quantitative questionnaire and the NOVA classification system (comprising 37 food groups and beverages). The study also collected and analyzed the blood parameters, as well as biochemical and anthropometric indices, for all participants.

**Results:**

Among the women included in the study, 22.9% had MHO phenotypes but 77.1% had MUHO phenotypes. A significant association between the third quartile of the NOVA classification system and the increased likelihood of having the MUHO phenotype was observed (OR = 1.40, 95% CI = 1.09–4.92, *p* = 0.04). Regarding the potential role of inflammatory markers, high-sensitivity C-reactive protein (hs-CRP) (*p* = 0.84), transforming growth factor-β (TGF-β) (*p* = 0.50), monocyte chemoattractant protein-1 (MCP-1) (*p* = 0.49), plasminogen activator inhibitor-1 (PAI-1) (*p* = 0.97), and homeostatic model assessment for insulin resistance (HOMA-IR) (*p* = 0.92) were found to be mediators.

**Conclusion:**

We observed a significant positive association between ultra-processed food (UPF) consumption and the MUHO phenotype in overweight and obese women. This association appeared to be mediated by some inflammatory markers, such as hs-CRP, TGF-β, MCP-1, PAI-1, and HOMA-IR. Additional studies are needed to validate these findings.

## 1 Introduction

Obesity is associated with mild inflammation, and it is a state or condition that actively participates in regulating both physiological and pathological inflammatory processes by releasing pro-inflammatory cytokines, such as C-reactive protein (CRP) (1, 2). This inflammatory response can significantly increase the risk of morbidity and mortality (1, 3–5). The etiology of obesity is influenced by factors such as genetics, gender, lifestyle, and dietary intake (6–9). It was projected that, by 2030, the number of overweight adults will reach 2.16 billion and the number of obese adults will be approximately 1.12 billion (10). A study conducted on a population of 5,607 individuals reported an overall obesity prevalence of 57.2%, which was higher among women compared to men (11). However, not all obese individuals experience metabolic dysfunction, particularly cardiometabolic issues (12). Recent definitions classify obese individuals into metabolically healthy obese (MHO) and metabolically unhealthy obese (MUHO) groups based on their metabolic status. The MHO group (with a prevalence of 10–34%) is more prevalent in women and comprises individuals with specific metabolic profiles, such as higher insulin sensitivity, a lower incidence of hypertension, and optimal inflammation levels (13–16). Various indicators and definitions have been used to determine the metabolic health status, and in this study, the Karelis criterion was employed to categorize individuals into either the MHO or MUHO group (14, 15, 17, 18).

Dietary intake plays a crucial role in the development of overweight and obesity, and numerous studies have highlighted the significant impact of dietary patterns on obesity phenotypes (8, 19–21). Over the past few decades, there has been a notable increase in the consumption of processed foods due to the increasing availability of industrially processed products (22). Consequently, studies have reported associations between the consumption of processed foods, such as sugar (23), sugar-sweetened beverages (SSB) (24), fast food (25), and obesity, as well as unhealthy metabolic phenotypes (26–28). The energy-dense high-saturated fatty acid (SFA) diet enhances insulin resistance (IR), obesity, and metabolic syndrome (29, 30). Literature shows that ultra-processed food (UPF) intake affects inflammatory markers, such as high-sensitivity C-reactive protein (hs-CRP), tumor necrosis factor-α (TNF-α), and interleukin-6 (IL-6) (31, 32). Several studies have been conducted on the relationship between inflammatory markers and obesity and how they activate the innate immune system in the adipose tissue, especially the visceral fat (33–35). However, regarding the relationship between UPF consumption and inflammatory markers, such as CRP, there are conflicting findings. In a cross-sectional study examining this relationship in both sexes, only a significant relationship was observed in women but not in men (36).

The NOVA classification system is a novel approach to assess the dietary patterns while grouping foods into four categories: unprocessed or minimally processed foods, processed culinary ingredients, processed foods, and UPFs (37). There was a significant association between UPF intake and an increased risk of chronic disorders such as diabetes and cardiovascular disease (CVD) (38–41). A prospective study in the Brazilian population showed more weight and waist circumference (WC) gains in the highest quartile of UPF consumption (42). Limited studies have reported an association between NOVA and increased inflammatory markers (31), which may be due to the consumption of SFAs, potatoes, hydrogenated fats, snacks, sugar, soft drinks, sweets, and desserts (43–46). However, there is currently a lack of studies exploring the relationship between UPF intake and inflammatory markers using the NOVA classification system among women. We aimed to investigate the mediating role of inflammatory markers in the relationship between the NOVA classification system and obesity phenotypes among women.

## 2 Subjects and methods

### 2.1 Participants

In this cross-sectional study, 221 overweight and obese women in the age range of 18–56 years participated. Participants with body mass index (BMI) between 25 kg/m^2^ and 29.9 kg/m^2^ were classified as overweight and those with BMI ≥30 kg/m^2^ as obese. Participants were selected from those who attended the health clinics of Tehran University of Medical Sciences (TUMS), Tehran Province, Tehran, Iran. Participants were included in the study after signing a written informed consent. The study protocol entered the practical phase after being approved by the ethics committee of TUMS (Ethics ID: IR.TUMS.MEDICINE.REC. 1399.165). Participants having any history of disease, i.e., diabetes mellitus, CVD, hypertension, acute or chronic inflammatory disease, impaired renal and liver function, thyroid disease, autoimmune disease, malignancy, those who were pregnant and lactating, smokers, those with a regular use of drugs, including birth control pills, and those who consumed alcohol or had drug abuse, were excluded from the study. In addition, participants who had an energy intake of less than 800 kcal/day and more than 4,200 kcal/day, as well as those who had 5% weight fluctuations over the last year, were excluded from the study (47).

### 2.2 Assessment of dietary intakes

The dietary intakes were collected using a 147-item semi-quantitative food frequency questionnaire (FFQ) by a trained nutritionist. This validated and reliable questionnaire of Iranian adults (48, 49) includes a list of common foods consumed by the Iranian community, along with their standard portion sizes. Frequency of intake of each food item was asked and recorded as per daily, weekly, monthly, or annual consumption. After calculating the total energy intake (kcal/day), the data were analyzed using Nutritionist IV software (version 7.0; N-Squared Computing, Salem, OR, United States) (50). The standard portion size and items reported based on the household scale were collected and converted to grams using the home scale guide. Thus, the equivalent of grams consumed for each item and for each subject was calculated (51).

### 2.3 NOVA classification

The NOVA classification was used to assess the amount of UPF intake (52). NOVA classifies a total of 37 food items and beverages obtained from the FFQs as UPFs. These UPFs are further divided into seven distinct food groups as follows (Supplementary Figure S1):

1. Group 1: Non-dairy drinks (industrial sweet drinks, cola, nectar, and coffee). Coffee torrefaction is often industrial in nature, and food technology specialists view it as a high-impact process whose elevated temperatures give rise to higher acrylamide levels (53).
2. Group 2: Dairy drinks [ice cream (pasteurized and unpasteurized), cocoa milk, and chocolate milk].
3. Group 3: Cakes and cookies [industrial bread, cakes, pancakes, noodles, toast, pasta, cookies, biscuits, and sweets (creamy and non-creamy)].
4. Group 4: Fast food and processed meats (pizza, burger, sausage, and bologna).
5. Group 5: Salty snacks (chips, puff pastry, and crackers).
6. Group 6: Oil and sauce (mayonnaise, ketchup, and margarine).
7. Group 7: Sweets (turmeric, nougat, chocolate, candy, stone candy, sweets, and jam) (54, 55). Candy and sweet ingredients that are used in Iran as UPFs include these items.

### 2.4 Measurement of biochemical parameters

The participants’ blood samples after 8–12 h overnight fasting were collected with standard methods at the Nutrition and Biochemistry Laboratory of the School of Nutritional Sciences and Dietetics, TUMS (53). Triglyceride (TG), total cholesterol (T-Chol), high-density lipoprotein cholesterol (HDL-C), low-density lipoprotein cholesterol (LDL-C), and fasting blood sugar (FBS) levels were measured using glycerol-3-phosphate oxidase phenol 4-amino antipyrine peroxidase (GPO-PAP), enzymatic endpoint, direct enzymatic clearance, and glucose oxidase phenol 4-amino, respectively. Pars Azmoon laboratory kits (Pars Azmoon Inc., Tehran, Iran) were used to assess the serum levels of TG, T-Chol, HDL-C, LDL-C, glucose, and insulin. IR (mIU/ml) was calculated through the homeostatic model assessment (HOMA-IR) according to the following equation: [fasting plasma glucose (mmol/L) × fasting plasma insulin (μIU/L)]/22.5 (56). The enzyme-linked immunosorbent assay (ELISA) technique was used to evaluate the levels of inflammatory biomarkers. Levels of hs-CRP, monocyte chemoattractant protein-1 (MCP-1) (ZellBio GmbH, Ulm, Germany), transforming growth factor-β (TGF-β) (Human TGF-β1 Quantikine ELISA Kit, R&D Systems, United States), plasminogen activator inhibitor-1 (PAI-1) (Human PAI-1*96 T ELISA Kit Crystal Company), galectin-3 (Gal-3), and interleukin-1β (IL-1β) were measured using an immunoturbidimetric test.

### 2.5 Anthropometry assessment

The body weight of the subjects was measured using a digital scale with a precision of 100 g. They were instructed to wear minimal clothing and no shoes during the measurements. Participant’s height was recorded in a standing position with the normal position of the shoulders, without shoes. BMI was calculated by dividing the weight (kg) by the square of the height (m^2^). We used a multi-frequency bioelectrical impedance analyzer [InBody 770 scanner (InBody Co., Ltd., Seoul, South Korea)] to assess the body composition, including the visceral fat level (VFL), body fat percentage (BF%), fat-free mass index (FFMI) and fat mass index (FMI), WC, waist-to-hip ratio (WHR), fat-free mass (FFM), fat trunk, and fat mass (FM). To record a more accurate result, participants were asked to be present with empty stomachs and bladders, without exercising before the session, and without their shoes, jackets, and coats. The output was printed out after 20 min of measurement.

### 2.6 Assessment of other variables

The assessment of physical activity (PA) levels was conducted using the International Physical Activity Short Form Questionnaire (IPAQ). This questionnaire was administered through interviews, with participants providing oral responses. PA was quantified as metabolic equivalent task minutes per week (MET-minutes/week) (57). Additionally, a demographic questionnaire was utilized to gather information on age, educational background, employment status, marital status, and economic status.

### 2.7 Definition of metabolic health and its components

The assessment of the metabolic health status in individuals was conducted using the Karelis criterion, which considers both insulin sensitivity and inflammatory profiles. According to the Karelis criterion, individuals are considered metabolically healthy if they meet four or more of the following five components: HOMA-IR ≤ 2.7, hs-CRP ≤ 3.0 mg/L, LDL-C ≤ 2.6 mmol/L without any treatment, HDL-C ≥ 1.3 mmol/L without any treatment, and TG ≤ 1.7 mmol/L (14).

### 2.8 Statistical analysis

Data analysis was conducted using SPSS version 26 (SPSS Inc.). The normality of data distribution was assessed using the Kolmogorov–Smirnov test. Continuous variables were reported as mean ± standard deviation (SD), and the categorical variables were reported as numbers and percentages. The mean and SD were reported by a one-way analysis of variance (ANOVA). Analysis of covariance (ANCOVA) was used to examine the relationship between demographic variables (age, height, weight, PA, inflammatory parameters, food groups, macronutrients, and micronutrients) and the NOVA classification system while controlling for confounding variables, including age, BMI, and PA factors. The chi-square test was utilized to analyze the frequency of categorical variables, including education, economic status, marital status, employment status, and the NOVA classification system. Binary logistic regression analysis was employed to evaluate the association between NOVA classification scores and MUHO, with odds ratios (ORs) and 95% confidence intervals (CIs) reported. The analysis was performed in three models: crude, model 1, and model 2. The crude model did not include any confounding factors, with model 1 adjusted for age, BMI, energy intake, and PA and model 2 adjusted for age, BMI, energy intake, PA, economic status, supplement use, educational background, and marital status. In addition, to evaluate the mediatory role, Barrett’s model was used, and inflammatory markers including hs-CRP, TGF-β, IL-1β, MCP-1, PAI-1, and HOMA-IR were included in the adjustment *models.* value of *p*s of <0.05 were considered to be statistically significant, and value of *p*s of 0.05, 0.06, and 0.07 were considered marginally statistically significant.

## 3 Results

### 3.1 Study population and general characteristics

A total of 221 women who were overweight or obese were included in the analysis. The participants had a mean (SD) age of 36.22 (8.54) years, a body weight of 79.58 (10.89) kg, and a BMI of 30.55 (3.590) kg/m^2^. Most participants were married (71.9%), employed (97.0%), and had a moderate economic status (42.4%). The mean (SD) values for the NOVA score, Karelis criterion score, and hs-CRP were 197.149 (136.80), 2.471 (1.11), and 4.532 (4.72) mg/L, respectively. Additionally, most participants (77.1%) were classified as MUHO.

### 3.2 General characteristics across quartiles of NOVA classification

Table 1 represents general characteristics and anthropometric variables across the quartiles of the NOVA classification. A significant difference was observed in mean age (*p* = 0.03), WHR (*p* = 0.02), and economic status (*p* = 0.01) in the crude model. However, after adjusting for confounding factors such as age, BMI, PA, and energy intake, no significant associations were found for these variables (*p* > 0.05). Other variables did not show any significant relationships among the NOVA quartiles (p > 0.05). Furthermore, after adjusting for confounders, a significant difference was observed in variables such as MCP-1 (*p* = 0.06), Gal-3 (*p* = 0.03), PAI-1 (*p* = 0.024), and supplement use (p = 0.03).

**Table 1:** General characteristics of the participants in the study among quartiles of the NOVA classification system in overweight and obese women (*N* = 221).

Variables		Quartiles of the NOVA classification system									
		Q1 (<109.41 gr) (*n* = 69)		Q2 (109.41–174.03 gr) (*n* = 51)		Q3 (>174.03–276.75 gr) (*n* = 51)		Q4 (>276.75 gr) (*n* = 50)		Value of *p*	Value of p*
		Mean ± SD		Mean ± SD		Mean ± SD		Mean ± SD			
Age (years)		37.28 ± 8.08		38.17 ± 8.45		35.30 ± 9.5		33.64 ± 8.28		**0.03**	0.40
Height (cm)		160.14 ± 5.86		161.89 ± 5.92		162.18 ± 4.86		161.36 ± 5.61		0.19	0.89
Weight (kg)		77.78 ± 10.10		80.87 ± 9.89		80.75 ± 11.25		78.84 ± 10.78		0.30	0.55
BMI (kg/m^2^)		30.38 ± 3.48		30.74 ± 3.08		30.82 ± 3.61		30.44 ± 4.17		0.89	0.84
Body composition variables											
BFM (kg)		32.34 ± 6.63		32.77 ± 6.58		34.38 ± 7.28		33.10 ± 8.41		0.48	0.71
FFM (kg)		45.54 ± 5.59		47.85 ± 5.34		46.90 ± 5.44		46.14 ± 4.87		0.11	0.64
FFMI (kg/m^2^)		19.65 ± 15.84		18.22 ± 1.16		17.78 ± 1.54		17.70 ± 1.35		0.59	0.51
FMI (kg/m^2^)		12.78 ± 2.80		12.52 ± 2.50		13.04 ± 2.58		12.80 ± 3.36		0.83	0.90
BF (%)		41.28 ± 4.76		40.39 ± 4.53		41.80 ± 4.65		40.67 ± 6.62		0.51	0.64
WHR		0.92 ± 0.05		0.94 ± 0.04		0.93 ± 0.050		0.92 ± 0.05		**0.02**	0.08
WC (cm)		96.70 ± 8.81		99.63 ± 8.55		99.55 ± 8.91		97.37 ± 10.15		0.20	0.33
Visceral fat area (cm^2^)		157.22 ± 33.44		159.88 ± 34.50		167.01 ± 32.98		154.08 ± 44.21		0.31	0.60
Visceral fat level		15.11 ± 3.14		15.33 ± 3.17		16.03 ± 2.91		18.74 ± 27.59		0.48	0.51
Fat trunk (kg)		15.83 ± 3.02		16.35 ± 3.04		16.79 ± 3.10		16.24 ± 4.16		0.48	0.73
Fat trunk percentage		306.31 ± 59.54		304.34 ± 59.47		314.24 ± 53.14		305.52 ± 73.48		0.84	0.70
Blood parameters											
FBS (mg/dl)		87.40 ± 11.58		88.43 ± 8.96		87.90 ± 8.38		86.12 ± 8.73		0.66	0.38
T-Chol (mg/dl)		188.14 ± 36.92		186.11 ± 35.07		184.19 ± 40.44		180.24 ± 31.52		0.69	0.56
TG (mg/dl)		118.97 ± 58.50		126.62 ± 65.34		119.70 ± 57.48		109.22 ± 51.07		0.52	0.22
HDL-C (mg/dl)		47.53 ± 10.97		46.11 ± 10.35		43.78 ± 11.96		46.16 ± 8.55		0.29	0.89
LDL-C (mg/dl)		99.59 ± 23.96		95.76 ± 25.09		89.70 ± 22.89		93.38 ± 23.85		0.15	0.13
Inflammatory parameters											
MCP-1 (mg/L)		61.46 ± 115.36		48.29 ± 96.50		58.24 ± 101.35		48.33 ± 62.31		0.87	**0.06**
Gal-3 (mg/L)		5.37 ± 9.85		3.61 ± 4.89		4.45 ± 9.12		3.19 ± 3.98		0.80	**0.03**
PAI-1 (mg/L)		20.34 ± 41.73		10.71 ± 15.35 ^a^		16.66 ± 27.00^a^		16.07 ± 23.36		0.53	**0.02**
IL-1β (mg/L)		2.61 ± 0.86		2.87 ± 1.20		2.70 ± 0.88		2.85 ± 0.88		0.79	0.23
TGF-β (mg/L)		76.15 ± 43.83		88.63 ± 75.33		66.75 ± 17.42		84.95 ± 45.02		0.27	0.25
Hs-CRP (mg/L)		3.80 ± 4.50		4.88 ± 5.03		5.01 ± 5.20		4.52 ± 4.34		0.51	0.18
PA (METs-minutes/week)		1068.25 ± 1231.22		922.10 ± 1347.43		960.84 ± 882.06		1030.38 ± 1055.67		0.91	0.85
HOMA-IR		3.51 ± 1.57		3.38 ± 1.02		3.36 ± 1.16		3.26 ± 1.22		0.77	0.82
Insulin (mg/L)		1.19 ± 0.18		1.21 ± 0.26		1.248 ± 0.268		1.223 ± 0.226		0.67	0.84
Categorical variables		*N*	%	*N*	%	*N*	%	*N*	%	*p*-value	*P*-value*
Education status	Illiterate	0	0	1	33.3	2	66.7	0	0	0.30	0.36
	Under diploma	5	19.2	9	34.6	7	26.9	5	19.2		
	Diploma	32	36.8	19	21.8	20	23.0	16	18.4		
	Bachelor’s degree and higher	31	30.1	22	21.4	22	21.4	28	27.2		
Economic status	Low level	17	31.5	17	31.5	11	20.4	9	16.7	**0.01**	0.47
	Moderate level	30	31.3	25	26.0	18	18.8	23	24.0		
	High level	20	35.1	6	10.5	15	26.3	16	28.1		
Marital status	Single	15	24.2	14	22.6	17	27.4	16	25.8	0.81	0.675
	Married	53	33.8	37	23.6	34	21.7	33	21.0		
Job status	Unemployed	1	100	0	0	0	0	0	0	0.70	0.54
	Employed	68	31.3	50	23.0	50	23.0	49	22.6		
Supplement	Yes	33	31.7	26	25	21	20.2	24	23.1	0.86	**0.03**
	No	22	31.9	11	15.9	17	24.6	19	27.5		

BMI, body mass index; BFM, body fat mass; FFM, fat-free mass; FFMI, fat-free mass index; FMI, fat-mass index; BF%, body fat percentage; WHR, waist to hip ratio; WC, waist circumference; FBS, fasting blood sugar; T-Chol, total cholesterol; TG, triglycerides; HDL-C, high-density lipoprotein; LDL-C, low-density lipoprotein; MCP-1, monocyte chemoattractant protein-1; Gal-3, galectin-3; PAI-1, plasminogen activator inhibitor-1; IL-1B, interleukin-1 beta; TGF-β, transforming growth factor-β; hs-CRP, high-sensitive C-reactive protein; HOMA-IR, homeostatic model assessment for insulin resistance; PA, physical activity. Mean ± SD resulted from NOVA for continuous variables, and *N* (%) resulted from Q square for categorical variables. *p*-value, *p*-value for crude model; *p*-value*, *p*-value adjusted for age, BMI, energy intake, and PA. BMI is considered as a collinear variable for body composition and anthropometric measurements. *p*-values of < 0.05 were considered statistically significant, and *p*-value of 0.05, 0.06, and 0.07 were considered marginally significant. *p*-values of < 0.05 were considered statistically significant, and *p*-value of 0.05, 0.06, and 0.07 were considered marginally significant.

### 3.3 Dietary intake according to the NOVA quartiles

Table 2 displays the dietary intakes of the subjects categorized into NOVA classification system quartiles. The mean differences in protein, carbohydrate, SFA, monounsaturated fatty acid (MUFA), polyunsaturated fatty acid (PUFA), linoleic acid, linolenic acid, vitamin A, vitamin C, vitamin E, vitamin B1, vitamin B2, vitamin B3, vitamin B5, vitamin B9, vitamin B12, biotin, calcium, iron, zinc, copper, manganese, selenium, chromium, fruit consumption, refined grain consumption, dairy consumption, and red meat intake were found to be significant among the NOVA quartiles in the crude model (*p* < 0.05). In the adjusted model, significant mean differences were observed for vitamin B6, total fiber intake, SSB consumption, processed food consumption, and vegetable consumption (*p* < 0.05).

**Table 2:** Dietary intakes according to NOVA classification system quartiles in study participants (*N* = 221).

Quartiles of the NOVA classification system						
Variables	Q1 (<109.41 gr) (*n* = 69)	Q2 (109.41–174.03 gr) (*n* = 51)	Q3(>174.03–276.75 gr) (*n* = 51)	Q4 (>276.75 gr) (*n* = 50)	*p*-value	*p*-value*
	Mean ± SD	Mean ± SD	Mean ± SD	Mean ± SD		
Energy (kcal/d)	2042.15 ± 525.15	2558.11 ± 601.62	2717.52 ± 667.68	3215.88 ± 692.95	**<0.001**	—
Macronutrients						
Protein (g/d)	72.97 ± 23.79	84.64 ± 23.96	92.15 ± 21.32	107.55 ± 31.84	**<0.001**	0.62
Carbohydrate (g/d)	290.05 ± 88.87	364.99 ± 106.52	387.58 ± 109.66	463.18 ± 109.93	**<0.001**	0.90
Total fat (g/d)	72.71 ± 21.78	92.73 ± 30.65	97.72 ± 29.43	114.57 ± 30.24	**<0.001**	0.87
Subgroups of fat						
Chol (mg/d)	210.23 ± 70.16	241.36 ± 99.33	281.51 ± 95.74	284.05 ± 125.08	**<0.001**	0.28
SFA (mg/d)	21.73 ± 7.31	26.97 ± 11.38	30.68 ± 11.61	33.40 ± 11.39	**<0.001**	0.55
MUFA (g/d)	24.62 ± 8.65	30.99 ± 11.55	32.32 ± 10.21	36.29 ± 10.05	**<0.001**	0.76
PUFA (g/d)	16.44 ± 7.37	20.21 ± 9.44	19.61 ± 7.69	23.11 ± 7.97	**<0.001**	0.75
Linoleic acid (g/d)	14.03 ± 6.96	17.59 ± 8.95	16.96 ± 7.35	19.99 ± 7.72	**0.001**	0.76
Linolenic acid (g/d)	1.06 ± 0.64	1.32 ± 0.69	1.20 ± 0.52	1.38 ± 0.57	**0.02**	**0.06**
EPA (mg/d)	0.03 ± 0.04	0.02 ± 0.02	0.03 ± 0.03	0.03 ± 0.04	0.69	0.28
DHA (mg/d)	0.11 ± 0.12	0.09 ± 0.07	0.10 ± 0.09	0.11 ± 0.14	0.72	0.32
TFA (mg/d)	0.001 ± 0.003	0.001 ± 0.002	<0.001 ± 0.001	0.001 ± 0.002	0.52	0.36
Micronutrients and Vitamins						
Vitamin A (RAE)	682.35 ± 348.71	745.75 ± 408.17	837.83 ± 451.93	914.70 ± 437.27	**0.01**	0.64
Β-carotene (mg/d)	4826.76 ± 3431.32	5223.90 ± 3596.27	5668.682 ± 4580.386	5779.42 ± 3317.88	0.49	0.29
Vitamin E (mg/d)	14.81 ± 7.81	16.55 ± 8.68	17.12 ± 8.56	19.10 ± 9.08	**<0.001**	0.99
Vitamin K (μg/d)	208.96 ± 151.93	217.04 ± 173.29	237.49 ± 326.88	218.36 ± 176.57	0.91	0.57
Vitamin C (μmol/L)	161.72 ± 100.81	189.06 ± 108.83	190.64 ± 107.73	256.60 ± 181.67	**0.001**	0.49
Vitamin B1(μg/d)	1.64 ± 0.47	2.09 ± 0.61	2.13 ± 0.60	2.46 ± 0.60	**<0.001**	0.40
Vitamin B2 (mg/d)	1.75 ± 0.59	2.10 ± 0.67	2.30 ± 0.64	2.77 ± 1.02	**<0.001**	0.37
Vitamin B3 (mg/d)	20.46 ± 6.68	24.02 ± 7.13	25.73 ± 6.83	30.85 ± 11.48	**<0.001**	0.66
Vitamin B5 (mg/d)	5.36 ± 1.71	6.29 ± 2.02	6.64 ± 1.83	7.97 ± 3.52	**<0.001**	0.77
Vitamin B6 (μg/d)	1.83 ± 0.57	2.14 ± 0.69	2.19 ± 0.61	2.56 ± 0.80	**<0.001**	**0.03**
Vitamin B9 (μg/d)	503.92 ± 153.74	624.28 ± 167.47	620.22 ± 156.23	693.46 ± 176.57	**<0.001**	0.08
Vitamin B12 (μg/d)	3.52 ± 1.74	4.04 ± 1.86	4.48 ± 1.42	5.77 ± 3.30	**<0.001**	0.14
Biotin (mg/day)	33.16 ± 14.32	36.52 ± 12.81	40.41 ± 14.85	46.64 ± 24.49	**<0.001**	0.64
Minerals						
Calcium (mg/d)	959.94 ± 357.03	1122.93 ± 396.33	1186.90 ± 372.93	1396.85 ± 430.55	**<0.001**	0.87
Iron (mg/d)	15.000 ± 4.83	18.77 ± 5.34	19.42 ± 5.50	22.53 ± 5.83	**<0.001**	0.37
Magnesium (mg/d)	387.74 ± 138.87	449.43 ± 138.89	490.02 ± 150.98	542.20 ± 129.30	**<0.001**	**0.03**
Zinc (mg/d)	10.51 ± 3.46	12.83 ± 4.36	13.74 ± 3.66	15.69 ± 4.15	**<0.001**	0.58
Copper (mg/d)	1.63 ± 0.55	2.01 ± 0.61	2.03 ± 0.59	2.47 ± 0.88	**<0.001**	0.69
Manganese (mg/d)	6.09 ± 3.15	7.02 ± 2.19	7.84 ± 3.02	7.90 ± 2.37	**0.001**	**0.06**
Selenium (mg/d)	94.19 ± 33.14	116.81 ± 32.45	131.17 ± 42.85	143.50 ± 41.41	**<0.001**	0.22
Chromium (mg/d)	0.09 ± 0.07	0.10 ± 0.07	0.13 ± 0.10	0.12 ± 0.08	**0.02**	0.11
Total fiber (g/d)	38.33 ± 17.28	47.24 ± 19.18	44.04 ± 16.96	50.89 ± 19.04	**0.002**	**0.01**
Food groups and other						
Whole grains (g/d)	8.20 ± 10.90	8.83 ± 11.35	6.37 ± 8.40	8.13 ± 12.38	0.69	0.22
Fruits (g/d)	419.20 ± 286.41	521.07 ± 327.56	542.11 ± 312.72	702.57 ± 423.02	**<0.001**	0.79
Vegetables (g/d)	445.71 ± 312.87	429.34 ± 237.19	401.70 ± 211.09	487.27 ± 288.93	0.44	**0.004**
Nuts (g/d)	12.52 ± 14.02	16.97 ± 22.36	14.83 ± 15.94	18.40 ± 17.19	0.28	0.21
Legumes (g/d)	51.06 ± 50.63	60.79 ± 43.43	54.81 ± 41.16	54.92 ± 36.56	0.69	0.32
Tea and coffee (ml/d)	680.71 ± 1225.10	663.14 ± 552.02	847.92 ± 493.90	887.19 ± 616.38	0.37	0.77
Refined grains (g/d)	318.91 ± 131.58	455.86 ± 247.79	461.25 ± 195.57	506.63 ± 268.62	**<0.001**	0.32
SSB (ml/d)	4.44 ± 8.74	8.84 ± 13.36	13.75 ± 18.98	64.78 ± 99.94	**<0.001**	**<0.001**
Dairy (ml/d)	320.36 ± 219.91	352.74 ± 218.64	386.42 ± 224.72	502.01 ± 300.83	**0.001**	0.55
Eggs (g/d)	18.90 ± 11.95	21.41 ± 12.05	25.11 ± 15.03	21.90 ± 16.78	0.12	0.29
Fish and seafood (g/d)	11.42 ± 12.08	9.77 ± 7.45	10.56 ± 8.26	12.87 ± 14.61	0.53	0.49
Meat (g/d)	52.70 ± 33.27	55.01 ± 33.36	64.77 ± 34.41	86.79 ± 74.83	**0.001**	0.34
White meat (g/d)	43.36 ± 34.19	36.83 ± 28.95	43.57 ± 29.18	56.22 ± 63.10	0.11	0.25
Red meat (g/d)	16.38 ± 12.88	21.25 ± 23.48	24.94 ± 19.98	29.91 ± 20.10	**0.002**	0.95
Processed food (g/d)	12.78 ± 12.74	16.58 ± 13.71	25.81 ± 21.02	46.86 ± 43.98	**<0.001**	**<0.001**
Caffeine (mg/d)	137.10 ± 245.06	133.02 ± 110.91	173.36 ± 98.99	182.87 ± 116.62	0.29	0.67

Chol, cholesterol; SFA, saturated fatty acid; MUFA, monounsaturated fatty acid; PUFA, polyunsaturated fatty acid; EPA, eicosapentaenoic acid; DHA, docosahexaenoic acid; TFA, trans-fatty acid; SSB, sugar-sweetened beverages. Values are represented as means ± SD. *p*-value: *p*-value crude; ANCOVA (*p*-value*) was performed to adjust potential confounding factors (energy intake). *p*-values < 0.05 were considered statistically significant, and *p*-values of 0.05, 0.06, and 0.07 were considered marginally significant.

### 3.4 Association between quartiles of NOVA classification and MUHO among overweight and obese women

The association between NOVA quartiles and MUHO in both the crude and adjusted models (model 1 and model 2) is represented in Table 3. The results of our study demonstrate a 77% increase in the odds of MUHO in the third quartile compared to the first quartile in the crude model (OR = 1.77, 95% CIs = 1.02, 4.33, *p* = 0.01). This association remained significant after adjustment in model 1 (controlling for age, BMI, energy intake, and PA) (OR = 1.95, 95% CIs = 1.65, 5.79, *p* = 0.04) and model 2 (controlling for age, BMI, energy intake, PA, economic status, supplement use, educational background, and marital status) (OR = 1.40, 95% CIs = 1.09, 4.92, *p* = 0.04).

**Table 3:** Association between NOVA classification score and MUHO among overweight and obese women (*N* = 221).

MUHO	Q1 (*n* = 69)	Q2 (*n* = 51)			Q3 (*n* = 51)			Q4 (*n* = 50)			*p*-value*
		OR	95% CI	*p*-value	OR	95% CI	*p*-value	OR	95% CI	*p*-value
Crude model	Ref.	1.55	0.65, 3.71	0.31	1.77	1.02, 4.33	**0.01**	0.97	0.43, 2.20	0.95	0.85
Model 1		1.56	0.57, 4.29	0.38	1.95	1.65, 5.79	**0.04**	1.08	0.34, 3.44	0.88	0.66
Model 2		1.43	0.43, 4.70	0.55	1.40	1.09, 4.92	**0.04**	1.27	0.31, 5.19	0.73	0.67

The first quartile of the Nova classification score was considered the reference group. Metabolic healthy was considered as the reference group. Model 1 was performed to adjust for potential confounding factors (age, BMI, energy intake, and PA). Model 2 was performed to adjust for potential confounding factors (age, BMI, energy intake, PA, economic status, supplement usage, education status, and marital status). OR: odds ratio; CIs: confidence intervals. *p*-values of < 0.05 were considered statistically significant, and *p*-value of 0.05, 0.06, and 0.07 were considered marginally significant.

### 3.5 Association of MUHO across the quartiles of NOVA, mediated by inflammatory markers

The relationship between MUHO and the NOVA quartiles, mediated by inflammatory markers, in overweight and obese women is shown in Table 4. The potential role of inflammatory markers, such as hs-CRP (*p* = 0.84), TGF-β (*p* = 0.50), MCP-1 (*p* = 0.49), PAI-1 (*p* = 0.97), and HOMA-IR (*p* = 0.92), was found to be that of mediators.

**Table 4:** Association of MUHO across the quartiles of NOVA, mediated by inflammatory markers.

Variables	Q1 (*n* = 69)	Q2 (*n* = 51)			Q3 (*n* = 51)			Q4 (*n* = 50)			OR	95% CI	*P*-value	OR	95% CI	*P*-value	OR	95% CI	*P*-value
Metabolic unhealthy	Ref.									
hs-CRP (mg/L)		1.21	0.30, 4.92	0.78	0.86	0.20, 3.67	0.84	0.72	0.12, 4.007	0.70
TGF-β (mg/L)		1.47	0.28, 7.68	0.64	1.89	0.29, 12.39	0.50	0.66	0.10, 4.19	0.66
IL-1β (mg/L)		0.33	0.02, 5.10	0.43	0.09	0.001, 0.99	**0.02**	0.03	0.001, 1.37	**0.07**
MCP-1 (mg/L)		1.79	0.48, 6.63	0.38	1.58	0.42, 5.96	0.49	1.17	0.28, 4.89	0.82
PAI-1 (mg/L)		1.78	0.37, 8.48	0.46	1.03	0.20, 5.19	0.97	0.98	0.17, 5.60	0.98
HOMA-IR		0.97	0.21, 4.48	0.97	1.07	0.24, 4.67	0.92	1.19	0.22, 6.48	0.83

The first quartile of the Nova classification score was considered the reference group. Metabolic healthy was considered as the reference group. OR, odds ratio; CIs, confidence intervals; hs-CRP, high-sensitive C-reactive protein; TGF-β, transforming growth factor-β; IL-1B, interleukin-1beta; MCP-1, monocyte chemoattractant protein-1; PAI-1, plasminogen activator inhibitor-1; HOMA-IR, homeostatic model assessment for insulin resistance. *p*-values of < 0.05 were considered statistically significant, and *p*-value of 0.05, 0.06, and 0.07 were considered marginally significant.

## 4 Discussion

The current study investigated the associations between the mediating role of inflammatory markers and the relationship between the NOVA classification system and obesity phenotypes in obese and overweight adult women.

There was a positive association observed between the third quartile of the NOVA classification system and an increased likelihood of having the MUHO phenotype in women. Inflammatory markers such as hs-CRP, TGF-β, MCP-1, PAI-1, and HOMA-IR showed their mediatory role in Q3. This finding is consistent with a study by Yu et al., which found that the visceral adipose tissue (VAT) is independently associated with various inflammatory markers. Specifically, markers such as white blood cell (WBC) count and hs-CRP showed stronger correlations with VAT compared to other markers such as the neutrophil–lymphocyte ratio (NLR) and platelet–lymphocyte ratio (PLR) (58). Furthermore, another study reported a significant negative association between adherence to a healthy plant-based diet index (hPDI) and the MUHO phenotype among overweight and obese Iranian women. This association was found to be mediated by factors such as TGF-β, IL-1β, and MCP-1 (59). Hosseininasab et al. indicated that there is a significant association between UPF consumption and TGF-β, atherogenic coefficient (AC), VFL, and the quantitative insulin sensitivity check index (QUICKI) (60). In a systematic review and meta-analysis of 43 observational studies, there was an association between intake of UPF and an increased risk of obesity, overweight, abdominal obesity, and metabolic syndrome (61). The higher consumption of dairy products, tea, and coffee compared to fast foods decreased the risk of developing an unhealthy phenotype (62). In a systematic review and meta-analysis study, there was an association between increased UPF consumption and a cardiometabolic risk profile (63). Exposure to bisphenol, an industrial chemical used in the plastic packaging of some UPFs, is related to an increased risk of cardiometabolic disease (64).

UPF intake must be cautiously adopted given the high proportion of artificial ingredients in their formulations (37), which are potentially harmful to human health, especially when consumed in excess. Excessive consumption of these foods is related to an increase in the occurrence of NCDs (37, 65). Emerging evidence indicates that the consumption of UPF products contributes to an unhealthy dietary pattern (26). Furthermore, additives present in foods such as emulsifiers and non-caloric artificial sweeteners have been linked to various chronic disorders such as systemic inflammation, endothelial dysfunction, and disrupted immune response (66, 67). A study conducted in the United States revealed that UPF consumption was associated with increased exposure to phthalates (68), which have been suggested to be linked to obesity (69). Evidence suggests that abdominal obesity’s origin can be associated with UPF intake, which delays satiety signals (37). UPF can also increase *ad libitum* energy intake by approximately 500 kcal/day, leading to higher weight gain compared to a minimally processed diet (70). Carrageenan, one of the commonly used additives, has been implicated in promoting IR and inhibiting insulin signaling in mice (71, 72), which may contribute to weight gain (73). On the other hand, a minimally processed diet has been shown to result in higher levels of peptide YY (an appetite suppressant hormone) compared to the UPF diet (70). Certain emulsifiers (such as carboxymethyl cellulose and polysorbate-80) have induced metabolic disturbances, alterations in the gut microbiota, and low-grade inflammation in mice (74).

The present study has its strengths. First, it is the first study to investigate the mediatory role of various inflammatory markers on the relationship between the NOVA classification system and obesity phenotypes among obese and overweight adult women. Second, the assessment of dietary intake was conducted using a validated and reliable questionnaire. Third, confounding factors were adjusted in the statistical models.

However, there are several limitations to be discussed. The observational design of the study restricts the ability to establish causality of associations. Nevertheless, an observational design is the most suitable approach to investigate these types of associations, as conducting longitudinal studies involving interventions targeting UPF intake could pose health risks and ethical concerns. Furthermore, the results cannot be extrapolated to the general population as the participants included in the study were overweight/obese Iranian women. In addition, the assessment of food intake using a FFQ is susceptible to measurement bias. Nevertheless, FFQs have been widely used as a tool in epidemiological studies since the 1990s (75). Finally, despite considering potential confounders, the possibility of residual confounding factors influencing the results cannot be entirely ruled out.

## 5 Conclusion

In conclusion, our findings indicate a significant positive association between the consumption of UPFs and the MUHO phenotype in overweight and obese women residing in Iran. This association appears to be mediated by inflammatory markers such as MCP-1, PAI-1, hs-CRP, TGF-β, and HOMA-IR. The results of this study suggest that UPF consumption may have detrimental effects on obesity-related characteristics and, consequently, on NCDs. The role of diet and nutritional status is crucial for improving human health. Identifying unhealthy dietary patterns and their association with obesity is essential for preventing chronic diseases and enhancing public health strategies. Further research is warranted to confirm and expand upon our findings.

## Data availability statement

The raw data supporting the conclusions of this article will be made available by the authors, without undue reservation.

## Ethics statement

Ethics approval for the study protocol was confirmed by The Human Ethics Committee of TUMS (Ethics Number: ID: IR. TUMS.MEDICINE. REC. 1399.165). All participants signed a written informed consent approved by the Ethics committee. The studies were conducted in accordance with the local legislation and institutional requirements. The participants provided their written informed consent to participate in this study.

## Author contributions

MH and DH wrote the manuscript. FS performed the statistical analyzes. DH, YA, and SH revised the manuscript and improved its language. KM had full access to all the data in the study and took responsibility for the integrity and accuracy of the data. All authors have read and approved the final manuscript.

## Figures and Tables

**Table:** 

ANCOVA	Analysis of covariance
BF%	Body fat percentage
BMI	Body mass index
CVD	Cardiovascular disease
ELISA	Enzyme-linked immunosorbent assay
FBS	Fasting blood sugar
FFM	Fat-free mass
FFMI	Fat-free mass index
FFQ	Food Frequency Questionnaire
FMI	Fat mass index
FM	Fat mass
Gal-3	Galectin-3
HDL-c	High-density lipoprotein cholesterol
hs-CRP	High-sensitivity C-reactive protein
HOMA-IR	Homeostatic model assessment for insulin resistance
IR	Insulin resistance
LDL-c	Low-density lipoprotein cholesterol
MCP-1	Monocyte chemoattractant protein-1
MUHO	Metabolically unhealthy obese
MHO	Metabolically healthy obese
PAI-1	Plasminogen activator inhibitor-1
IL-1β	Interleukin-1beta
IL-6	Interleukin-6
TGF-β	Transforming growth factor-β
TG	Triglyceride
T-Chol	Total cholesterol
UPF	Ultra-processed food
WBC	White blood cells
WC	Waist circumference
WHR	Waist-to-hip ratio
OR	Odds ratio
CI	Confidence interval
VAT	Visceral adipose tissue

## References

[1] Feve B Bastard J-P Vidal H. Relationship between obesity, inflammation and insulin resistance: new concepts. C R Biol. (2006) 329:587–97. doi: 10.1016/j.crvi.2006.03.020 16860277

[2] Schrager MA Metter EJ Simonsick E Ble A Bandinelli S Lauretani F. Sarcopenic obesity and inflammation in the InCHIANTI study. J Appl Physiol. (2007) 102:919–25. doi: 10.1152/japplphysiol.00627.2006, PMID: 17095641 PMC2645665

[3] Krakauer NY Krakauer JC. A new body shape index predicts mortality hazard independently of body mass index. PLoS One. (2012) 7:e39504. doi: 10.1371/journal.pone.0039504, PMID: 22815707 PMC3399847

[4] Pi-Sunyer X. The medical risks of obesity. Postgrad Med. (2009) 121:21–33. doi: 10.3810/pgm.2009.11.2074, PMID: 19940414 PMC2879283

[5] Zhang Y Liu J Yao J Ji G Qian L Wang J. Obesity: pathophysiology and intervention. Nutrients. (2014) 6:5153–83. doi: 10.3390/nu6115153, PMID: 25412152 PMC4245585

[6] Cameron AJ Zimmet PZ Dunstan DW Dalton M Shaw JE Welborn TA. Overweight and obesity in Australia: the 1999–2000 Australian diabetes, obesity and lifestyle study (AusDiab). Med J Aust. (2003) 178:427–32. doi: 10.5694/j.1326-5377.2003.tb05283.x, PMID: 12720507

[7] Cecil JE Tavendale R Watt P Hetherington MM Palmer CNA. An obesity-associated FTO gene variant and increased energy intake in children. N Engl J Med. (2008) 359:2558–66. doi: 10.1056/NEJMoa0803839 19073975

[8] Habibi N Leemaqz SY-L Grieger JA. Modelling the impact of reducing ultra-processed foods based on the NOVA classification in Australian women of reproductive age. Nutrients. (2022) 14:1518. doi: 10.3390/nu14071518, PMID: 35406131 PMC9003044

[9] Seidell JC. Epidemiology of obesity. in Seminars in vascular medicine. Stuttgart: Thieme Medical Publishers, Inc. (2005). 10.1055/s-2005-871737 15968575

[10] Kelly T Yang W Chen CS Reynolds K He J. Global burden of obesity in 2005 and projections to 2030. Int J Obes. (2008) 32:1431–7. doi: 10.1038/ijo.2008.102, PMID: 18607383

[11] Pourfarzi F Sadjadi A Poustchi H Amani F. Prevalence of overweight and obesity in Iranian population: a population-based study in northwestern of Iran. J Public Health Res. (2022) 11:2475. doi: 10.4081/jphr.2021.2475 PMC8859716 34545736

[12] Pajunen P Kotronen A Korpi-Hyövälti E Keinänen-Kiukaanniemi S Oksa H Niskanen L. Metabolically healthy and unhealthy obesity phenotypes in the general population: the FIN-D2D survey. BMC Public Health. (2011) 11:1–8. doi: 10.1186/1471-2458-11-754 21962038 PMC3198943

[13] Bell JA Kivimaki M Hamer M. Metabolically healthy obesity and risk of incident type 2 diabetes: a meta-analysis of prospective cohort studies. Obes Rev. (2014) 15:504–15. doi: 10.1111/obr.12157, PMID: 24661566 PMC4309497

[14] Hinnouho G-M Czernichow S Dugravot A Batty GD Kivimaki M Singh-Manoux A. Metabolically healthy obesity and risk of mortality: does the definition of metabolic health matter? Diabetes Care. (2013) 36:2294–300. doi: 10.2337/dc12-1654, PMID: 23637352 PMC3714476

[15] Muñoz-Garach A Cornejo-Pareja I Tinahones FJ. Does metabolically healthy obesity exist? Nutrients. (2016) 8:320. doi: 10.3390/nu8060320, PMID: 27258304 PMC4924161

[16] Van Vliet-Ostaptchouk JV Nuotio ML Slagter SN Doiron D Fischer K Foco L. The prevalence of metabolic syndrome and metabolically healthy obesity in Europe: a collaborative analysis of ten large cohort studies. BMC Endocr Disord. (2014) 14:1–13. doi: 10.1186/1472-6823-14-9 24484869 PMC3923238

[17] Alam I Ng TP Larbi A. Does inflammation determine whether obesity is metabolically healthy or unhealthy? The aging perspective. Mediat Inflamm. (2012) 2012:1–14. doi: 10.1155/2012/456456, PMID: 23091306 PMC3471463

[18] Hwang LC Bai CH Sun CA Chen CJ. Prevalence of metabolically healthy obesity and its impacts on incidences of hypertension, diabetes and the metabolic syndrome in Taiwan. Asia Pac J Clin Nutr. (2012) 21:227–33. PMID: 22507609

[19] Da Silva Scaranni PO De Oliveira Cardoso L Griep RH Lotufo PA Barreto SM Da Fonseca MJ. Consumption of ultra-processed foods and incidence of dyslipidaemias: the Brazilian longitudinal study of adult health (ELSA-Brasil). Br J Nutr. (2023) 129:336–44. doi: 10.1017/S0007114522001131 35450540

[20] Sung H Park JM Oh SU Ha K Joung H. Consumption of ultra-processed foods increases the likelihood of having obesity in Korean women. Nutrients. (2021) 13:698. doi: 10.3390/nu13020698, PMID: 33671557 PMC7926298

[21] Vilela DL Fonseca PG Pinto SL Bressan J. Influence of dietary patterns on the metabolically healthy obesity phenotype: a systematic review. Nutr Metab Cardiovasc Dis. (2021) 31:2779–91. doi: 10.1016/j.numecd.2021.05.007, PMID: 34340900

[22] Lawrence MA Baker PI. Ultra-processed food and adverse health outcomes. BMJ. (2019) 2019:l2289. doi: 10.1136/bmj.l2289 31142449

[23] Basu S Yoffe P Hills N Lustig RH. The relationship of sugar to population-level diabetes prevalence: an econometric analysis of repeated cross-sectional data. PLoS One. (2013) 8:e57873. doi: 10.1371/journal.pone.0057873, PMID: 23460912 PMC3584048

[24] Malik VS Hu FB. The role of sugar-sweetened beverages in the global epidemics of obesity and chronic diseases. Nat Rev Endocrinol. (2022) 18:205–18. doi: 10.1038/s41574-021-00627-6, PMID: 35064240 PMC8778490

[25] Bahadoran Z Mirmiran P Azizi F. Fast food pattern and cardiometabolic disorders: a review of current studies. Health Promot Perspect. (2015) 5:231–40. doi: 10.15171/hpp.2015.028, PMID: 26933642 PMC4772793

[26] Matos RA Adams M Sabaté J. The consumption of ultra-processed foods and non-communicable diseases in Latin America. Front Nutr. (2021) 8:622714. doi: 10.3389/fnut.2021.622714, PMID: 33842521 PMC8024529

[27] Moradi S Entezari MH Mohammadi H Jayedi A Lazaridi AV Kermani MH. Ultra-processed food consumption and adult obesity risk: a systematic review and dose-response meta-analysis. Crit Rev Food Sci Nutr. (2022) 63:249–60. doi: 10.1080/10408398.2021.1946005 34190668

[28] Neri D Martínez-Steele E Khandpur N Levy R. Associations between ultra-processed foods consumption and indicators of adiposity in US adolescents: cross-sectional analysis of the 2011-2016 National Health and nutrition examination survey. J Acad Nutr Diet. (2022) 122:1474–1487.e2. doi: 10.1016/j.jand.2022.01.005 35051632

[29] Feskens EJ Virtanen SM Räsänen L Tuomilehto J Stengård J Pekkanen J. Dietary factors determining diabetes and impaired glucose tolerance: a 20-year follow-up of the Finnish and Dutch cohorts of the seven countries study. Diabetes Care. (1995) 18:1104–12. doi: 10.2337/diacare.18.8.1104, PMID: 7587845

[30] Storlien L Baur LA Kriketos AD Pan DA Cooney GJ Jenkins AB. Dietary fats and insulin action. Diabetologia. (1996) 39:621–31. doi: 10.1007/BF00418533 8781757

[31] Dos Santos Martins GM França AK Viola PC Carvalho CA Marques KD Santos AM. Intake of ultra-processed foods is associated with inflammatory markers in Brazilian adolescents. Public Health Nutr. (2022) 25:591–9. doi: 10.1017/S1368980021004523, PMID: 34726140 PMC9991817

[32] Stanimirovic J Radovanovic J Banjac K Obradovic M Essack M Zafirovic S. Role of C-reactive protein in diabetic inflammation. Mediat Inflamm. (2022) 2022:1–15. doi: 10.1155/2022/3706508, PMID: 35620114 PMC9129992

[33] Fontana L Eagon JC Trujillo ME Scherer PE Klein S. Visceral fat adipokine secretion is associated with systemic inflammation in obese humans. Diabetes. (2007) 56:1010–3. doi: 10.2337/db06-1656 17287468

[34] Konieczna J Morey M Abete I Bes-Rastrollo M Ruiz-Canela M Vioque J. Contribution of ultra-processed foods in visceral fat deposition and other adiposity indicators: prospective analysis nested in the PREDIMED-plus trial. Clin Nutr. (2021) 40:4290–300. doi: 10.1016/j.clnu.2021.01.019 33610419

[35] Poti JM Braga B Qin B. Ultra-processed food intake and obesity: what really matters for health–processing or nutrient content? Curr Obes Rep. (2017) 6:420–31. doi: 10.1007/s13679-017-0285-4, PMID: 29071481 PMC5787353

[36] Lopes AE Araújo LF Levy RB Barreto SM Giatti L. Association between consumption of ultra-processed foods and serum C-reactive protein levels: cross-sectional results from the ELSA-Brasil study. Sao Paulo Med J. (2019) 137:169–76. doi: 10.1590/1516-3180.2018.0363070219 31314878 PMC9721234

[37] Monteiro CA Cannon G Moubarac JC Levy RB Louzada MLC Jaime PC. The UN decade of nutrition, the NOVA food classification and the trouble with ultra-processing. Public Health Nutr. (2018) 21:5–17. doi: 10.1017/S1368980017000234, PMID: 28322183 PMC10261019

[38] Llavero-Valero M Escalada-San Martín J Martínez-González MA Basterra-Gortari FJ de la Fuente-Arrillaga C Bes-Rastrollo M. Ultra-processed foods and type-2 diabetes risk in the SUN project: a prospective cohort study. Clin Nutr. (2021) 40:2817–24. doi: 10.1016/j.clnu.2021.03.039, PMID: 33933748

[39] Martinez-Perez C San-Cristobal R Guallar-Castillon P Martínez-González MÁ Salas-Salvadó J Corella D. Use of different food classification systems to assess the association between ultra-processed food consumption and cardiometabolic health in an elderly population with metabolic syndrome (PREDIMED-plus cohort). Nutrients. (2021) 13:2471. doi: 10.3390/nu13072471, PMID: 34371982 PMC8308804

[40] Srour B Fezeu LK Kesse-Guyot E Allès B Méjean C Andrianasolo RM. Ultra-processed food intake and risk of cardiovascular disease: prospective cohort study (NutriNet-Santé). BMJ. (2019) 365:l1451. doi: 10.1136/bmj.l1451 31142457 PMC6538975

[41] Srour B Fezeu LK Kesse-Guyot E Allès B Debras C Druesne-Pecollo N. Ultraprocessed food consumption and risk of type 2 diabetes among participants of the NutriNet-Santé prospective cohort. JAMA Intern Med. (2020) 180:283–91. doi: 10.1001/jamainternmed.2019.5942, PMID: 31841598 PMC6990737

[42] Canhada SL Luft VC Giatti L Duncan BB Chor D Fonseca MJM. Ultra-processed foods, incident overweight and obesity, and longitudinal changes in weight and waist circumference: the Brazilian longitudinal study of adult health (ELSA-Brasil). Public Health Nutr. (2020) 23:1076–86. doi: 10.1017/S1368980019002854, PMID: 31619309 PMC7282862

[43] Arya S Isharwal S Misra A Pandey RM Rastogi K Vikram NK. C-reactive protein and dietary nutrients in urban Asian Indian adolescents and young adults. Nutrition. (2006) 22:865–71. doi: 10.1016/j.nut.2006.05.002, PMID: 16829027

[44] Esmaillzadeh A Kimiagar M Mehrabi Y Azadbakht L Hu FB Willett WC. Dietary patterns and markers of systemic inflammation among Iranian women. J Nutr. (2007) 137:992–8. doi: 10.1093/jn/137.4.992, PMID: 17374666

[45] Khayyatzadeh SS Bagherniya M Fazeli M Khorasanchi Z Bidokhti MS Ahmadinejad M. A Western dietary pattern is associated with elevated level of high sensitive C-reactive protein among adolescent girls. Eur J Clin Investig. (2018) 48:e12897. doi: 10.1111/eci.12897, PMID: 29377099

[46] Schulze MB Hoffmann K Manson JAE Willett WC Meigs JB Weikert C. Dietary pattern, inflammation, and incidence of type 2 diabetes in women. Am J Clin Nutr. (2005) 82:675–84. doi: 10.1093/ajcn/82.3.675 16155283 PMC2563043

[47] Mirzababaei A Sajjadi SF Ghodoosi N Pooyan S Arghavani H Yekaninejad MS. Relations of major dietary patterns and metabolically unhealthy overweight/obesity phenotypes among Iranian women. Diabetes Metab Syndr Clin Res Rev. (2019) 13:322–31. doi: 10.1016/j.dsx.2018.09.012, PMID: 30641720

[48] Mirmiran P Hosseini-Esfahanil F Jessri M Mahan LK Shiva N Azizis F. Does dietary intake by Tehranian adults align with the 2005 dietary guidelines for Americans? Observations from the Tehran lipid and glucose study. J Health Popul Nutr. (2011) 29:39–52. doi: 10.3329/jhpn.v29i1.7564, PMID: 21528789 PMC3075058

[49] Rezazadeh A Rashidkhani B. The association of general and central obesity with major dietary patterns of adult women living in Tehran, Iran. J Nutr Sci Vitaminol. (2010) 56:132–8. doi: 10.3177/jnsv.56.132, PMID: 20495295

[50] Computing NS. *Nutritionist IV: Diet analysis and nutrition evaluation*. (1994).

[51] Ghaffarpour M. Houshiar-Rad A. Kianfar H., The manual for household measures, cooking yields factors and edible portion of foods. 7. Tehran: Nashre Olume Keshavarzy 42–58. (1999).

[52] Moubarac J-C Parra DC Cannon G Monteiro CA. Food classification systems based on food processing: significance and implications for policies and actions: a systematic literature review and assessment. Curr Obes Rep. (2014) 3:256–72. doi: 10.1007/s13679-014-0092-0 26626606

[53] Rattanarat P Chindapan N Devahastin S. Comparative evaluation of acrylamide and polycyclic aromatic hydrocarbons contents in Robusta coffee beans roasted by hot air and superheated steam. Food Chem. (2021) 341:128266. doi: 10.1016/j.foodchem.2020.128266 33035858

[54] Edalati S Bagherzadeh F Asghari Jafarabadi M Ebrahimi-Mamaghani M. Higher ultra-processed food intake is associated with higher DNA damage in healthy adolescents. Br J Nutr. (2021) 125:568–76. doi: 10.1017/S0007114520001981, PMID: 32513316

[55] Bahrampour N Shiraseb F Noori S Clark CCT Mirzaei K. Is there any putative mediatory role of inflammatory markers on the association between ultra-processed foods and resting metabolic rate? Front Nutr. (2022) 9:9. doi: 10.3389/fnut.2022.932225 PMC9606709 36313082

[56] Tanabe N Saito K Yamada Y Takasawa T Seki N Suzuki H. Risk assessment by post-challenge plasma glucose, insulin response ratio, and other indices of insulin resistance and/or secretion for predicting the development of type 2 diabetes. Intern Med. (2009) 48:401–9. doi: 10.2169/internalmedicine.48.1325 19293537

[57] Craig CL Marshall AL Sjöström M Bauman AE Booth ML Ainsworth BE. International physical activity questionnaire: 12-country reliability and validity. Med Sci Sports Exerc. (2003) 35:1381–95. doi: 10.1249/01.MSS.0000078924.61453.FB, PMID: 12900694

[58] Yu J-Y Choi WJ Lee HS Lee JW. Relationship between inflammatory markers and visceral obesity in obese and overweight Korean adults: an observational study. Medicine. (2019) 98:e14740. doi: 10.1097/MD.0000000000014740 30817629 PMC6831265

[59] Mohamadi A Shiraseb F Mirzababaei A Hosseininasab D Rasaei N Clark CCT. Circulating inflammatory markers may mediate the relationship between healthy plant-based diet and metabolic phenotype obesity in women: a cross-sectional study. Int J Clin Pract. (2022) 2022:1–12. doi: 10.1155/2022/8099382, PMID: 35685490 PMC9159206

[60] Hosseininasab D Shiraseb F Noori S Jamili S Mazaheri-Eftekhar F Dehghan M. The relationship between ultra-processed food intake and cardiometabolic risk factors in overweight and obese women: a cross-sectional study. Front Nutr. (2022) 9:945591. doi: 10.3389/fnut.2022.945591, PMID: 36017229 PMC9396040

[61] Lane MM Davis JA Beattie S Gómez-Donoso C Loughman A O'Neil A. Ultraprocessed food and chronic noncommunicable diseases: a systematic review and meta-analysis of 43 observational studies. Obes Rev. (2021) 22:e13146. doi: 10.1111/obr.13146, PMID: 33167080

[62] Mirmiran P Moslehi N Hosseinpanah F Sarbazi N Azizi F. Dietary determinants of unhealthy metabolic phenotype in normal weight and overweight/obese adults: results of a prospective study. Int J Food Sci Nutr. (2020) 71:891–901. doi: 10.1080/09637486.2020.1746955, PMID: 32237941

[63] Pagliai G Dinu M Madarena MP Bonaccio M Iacoviello L Sofi F. Consumption of ultra-processed foods and health status: a systematic review and meta-analysis. Br J Nutr. (2021) 125:308–18. doi: 10.1017/S0007114520002688, PMID: 32792031 PMC7844609

[64] Rancière F Lyons JG Loh VHY Botton J Galloway T Wang T. Bisphenol a and the risk of cardiometabolic disorders: a systematic review with meta-analysis of the epidemiological evidence. Environ Health. (2015) 14:1–23. doi: 10.1186/s12940-015-0036-5 26026606 PMC4472611

[65] Steele EM Juul F Neri D Rauber F Monteiro CA. Dietary share of ultra-processed foods and metabolic syndrome in the US adult population. Prev Med. (2019) 125:40–8. doi: 10.1016/j.ypmed.2019.05.004, PMID: 31077725

[66] Chassaing B Koren O Goodrich JK Poole AC Srinivasan S Ley RE. Dietary emulsifiers impact the mouse gut microbiota promoting colitis and metabolic syndrome. Nature. (2015) 519:92–6. doi: 10.1038/nature14232, PMID: 25731162 PMC4910713

[67] Martino JV Van Limbergen J Cahill LE. The role of carrageenan and carboxymethylcellulose in the development of intestinal inflammation. Front Pediatr. (2017) 5:96. doi: 10.3389/fped.2017.00096, PMID: 28507982 PMC5410598

[68] Buckley JP Kim H Wong E Rebholz CM. Ultra-processed food consumption and exposure to phthalates and bisphenols in the US National Health and nutrition examination survey, 2013–2014. Environ Int. (2019) 131:105057. doi: 10.1016/j.envint.2019.105057, PMID: 31398592 PMC6728187

[69] Vafeiadi M Myridakis A Roumeliotaki T Margetaki K Chalkiadaki G Dermitzaki E. Association of early life exposure to phthalates with obesity and cardiometabolic traits in childhood: sex specific associations. Front Public Health. (2018) 6:327. doi: 10.3389/fpubh.2018.00327, PMID: 30538977 PMC6277685

[70] Hall KD Ayuketah A Brychta R Cai H Cassimatis T Chen KY. Ultra-processed diets cause excess calorie intake and weight gain: an inpatient randomized controlled trial of ad libitum food intake. Cell Metab. (2019) 30:67–77.e3. e3. doi: 10.1016/j.cmet.2019.05.008, PMID: 31105044 PMC7946062

[71] Bhattacharyya S Feferman L Tobacman JK. Carrageenan inhibits insulin signaling through GRB10-mediated decrease in Tyr (P)-IRS1 and through inflammation-induced increase in Ser (P) 307-IRS1. J Biol Chem. (2015) 290:10764–74. doi: 10.1074/jbc.M114.630053 25784556 PMC4409242

[72] Bhattacharyya S O-Sullivan I Katyal S Unterman T Tobacman JK. Exposure to the common food additive carrageenan leads to glucose intolerance, insulin resistance and inhibition of insulin signalling in HepG2 cells and C57BL/6J mice. Diabetologia. (2012) 55:194–203. doi: 10.1007/s00125-011-2333-z 22011715

[73] Kahn SE Hull RL Utzschneider KM. Mechanisms linking obesity to insulin resistance and type 2 diabetes. Nature. (2006) 444:840–6. doi: 10.1038/nature05482 17167471

[74] Roca-Saavedra P Mendez-Vilabrille V Miranda JM Nebot C Cardelle-Cobas A Franco CM. Food additives, contaminants and other minor components: effects on human gut microbiota—a review. J Physiol Biochem. (2018) 74:69–83. doi: 10.1007/s13105-017-0564-2 28488210

[75] Shim J-S Oh K Kim HC. Dietary assessment methods in epidemiologic studies. Epidemiol Health. (2014) 36:36. doi: 10.4178/epih/e2014009 PMC4154347 25078382

