# Supplementary figures and images for: The mediatory role of inflammatory markers on the relationship between the NOVA classification system and obesity phenotypes among obese and overweight adult women: a cross-sectional study
Source: Front Nutr. 2023 Dec 11;10:1226162. doi: 10.3389/fnut.2023.1226162 (PMC10754978; doi:10.3389/fnut.2023.1226162)

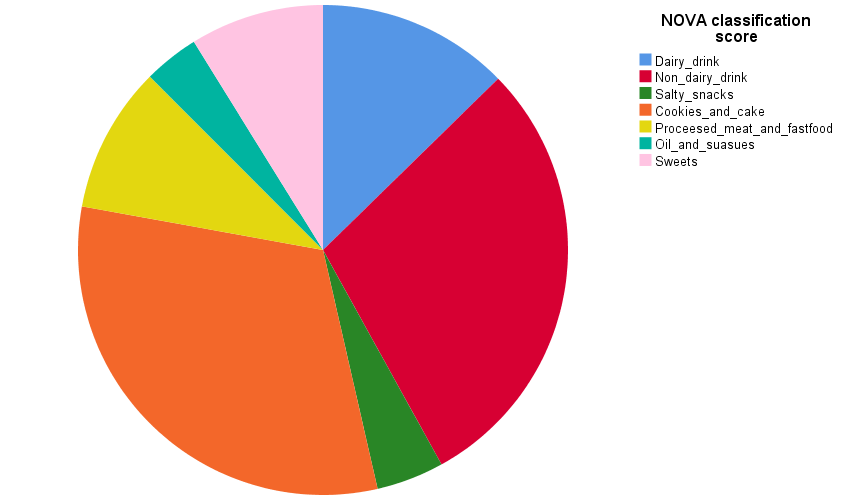


**Supplementary figure 1:** The percentage of components of NOVA classification score in study.

Supplement: Supplementary file 1 [file Data_Sheet_1.docx]
